# Sustained Ventricular Tachycardia and Cardiogenic Shock due to Scorpion Envenomation

**DOI:** 10.1155/2014/251870

**Published:** 2014-02-13

**Authors:** Carlos Henrique Miranda, Karina Tozatto Maio, Henrique Turin Moreira, Marcos Moraes, Viviane Imaculada do Carmo Custodio, Antonio Pazin-Filho, Palmira Cupo

**Affiliations:** ^1^Division of Emergency Medicine, Department of Internal Medicine, São Paulo University, Medical School at Ribeirão Preto, Rua Bernardino de Campos 1000, 14015-130 Ribeirão Preto, SP, Brazil; ^2^Department of Pediatrics, São Paulo University, Medical School at Ribeirão Preto, Rua Bernardino de Campos 1000, 14015-130 Ribeirão Preto, SP, Brazil

## Abstract

We describe a case of severe scorpion envenomation in an adult patient, with the presence of very rapid sustained ventricular tachycardia followed by cardiogenic shock, which was reversed by scorpion antivenom administration. Scorpion venom causes cardiac changes that can lead to an environment favoring arrhythmogenesis.

## 1. Introduction

Scorpion stings occur in many areas of the world, mainly in rural regions of countries with a hot climate [1]. A total of 51,457 scorpion stings were reported in Brazil during the year of 2010 [2]. Because of its frequency, the World Health Organization considers scorpion envenomation as the second most important type of poisoning caused by animals around the world [3].

The majority of these incidents are not serious, with local pain being the only clinical manifestation. However, severe complications such as acute pulmonary edema and cardiogenic shock can occur, mainly in children [1, 4].

We report here an unusual presentation of a case of severe scorpion envenoming complicated by acute pulmonary edema and cardiogenic shock in an adult patient, with the presence of very rapid sustained ventricular tachycardia during the early phase.

## 2. Case Presentation

**Figure 1:**
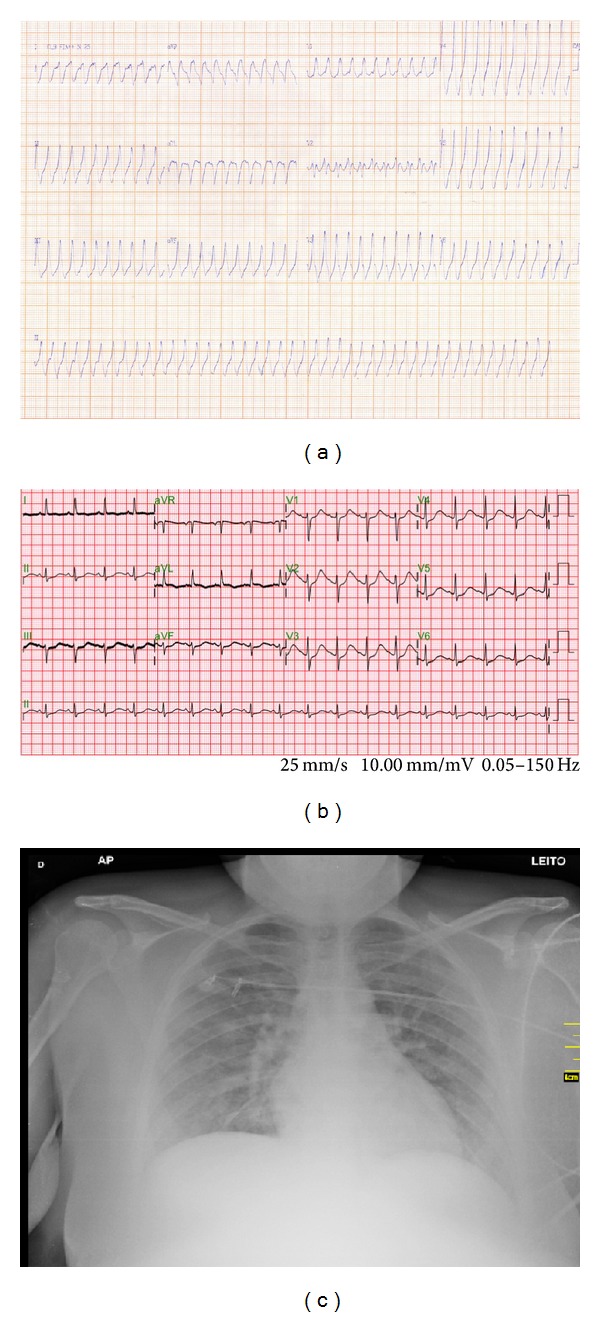
Electrocardiogram of a 25-year-old woman showing an episode of sustained ventricular tachycardia (SVT) with very fast heart rate (approximately 300 bpm) after scorpion envenomation (a). Upon admission to the hospital, the electrocardiogram showed sinus tachycardia associated with QTc interval prolongation (550 ms) after SVT reversal (b). A chest radiograph shows pulmonary edema with a normal cardiac area (c).

A 25-year-old woman with no previous disease was admitted to an emergency department after being stung in a toe by an unidentified animal when she was walking at night in a place infested with scorpions in an urban area near the local cemetery. A few minutes after the sting, she had important local pain and paresthesia of her entire left leg associated with several episodes of emesis, profuse sweating, mild respiratory distress, hypertension, and tachycardia. She first sought primary medical care, with clinical examination showing blood pressure of 220 × 110 mm Hg and a heart rate of 156 bpm. The electrocardiogram showed sustained ventricular tachycardia with very fast heart rate of approximately 300 bpm associated with a right bundle branch block pattern (Figure 1(a)). Amiodarone was initiated and the sinus rhythm was restored after some minutes, with the patient then being taken to our hospital. During this initial evaluation, no analgesics or antihypertensive agents were administered.

Upon hospital admission, moderate respiratory distress was observed with a respiratory rate of 30 incursions per minute and oxygen saturation of 89% in room air associated with bilateral rales compatible with acute pulmonary edema. The patient's heart rate was 110 bpm and her blood pressure was 110 × 70 mm Hg. The electrocardiogram showed sinus tachycardia associated with QTc interval prolongation (Figure 1(b)) and chest radiographs demonstrated bilateral fluffy shadows compatible with pulmonary edema and a normal cardiac area (Figure 1(c)). Laboratory tests showed hyperglycemia, hypokalemia, important metabolic acidosis with lactate elevation, leukocytosis, and troponin I elevation (Table 1). Because of this severe presentation, scorpion antivenom (8 ampoules, Butantan Institute, São Paulo) was administered intravenously about two hours and thirty minutes after the incident and local pain was relieved with lidocaine infiltration.

During the first hour after admission, she presented important hypotension (62 × 23 mm Hg), poor peripheral perfusion, and worsening of respiratory distress. A diagnosis of cardiogenic shock was made at that time and dobutamine was started. After this intervention, the hemodynamic status of the patient improved.

On the second day, echocardiography showed important depression of the left ventricular ejection fraction (30%) and severe hypokinesia in wall motion, except for the apical region, which was hyperkinetic. At that time, her clinical condition improved considerably and dobutamine was discontinued.

On the third day, the echocardiography was repeated and showed a mild depression of left ventricular ejection fraction (41%) associated with hypokinesia only in the basal segments. She received antiremodeling drugs consisting of an angiotensin-converting enzyme inhibitor (captopril) and a beta blocker (carvedilol).

On the sixth day, the patient was discharged and instructed to continue to take the above drugs. One month later, she was evaluated at an outpatient service and was found to be asymptomatic. Echocardiography was repeated and found to have fully normalized, and the two drugs were discontinued.

## 3. Discussion

We describe here a case of scorpion envenomation complicated by very rapid sustained ventricular tachycardia followed by cardiogenic shock. In this report we wish to emphasize two points. The first and more important point is the early presence of this life-threatening ventricular arrhythmia in this setting and the second is the severe clinical manifestation in an adult patient.

The major point of this report is the presence of monomorphic sustained ventricular tachycardia during the early phase of envenomation. Intense adrenergic activation with release of catecholamine in the circulation during envenomation might have a pivotal function since sympathetic overstimulation is a well-recognized factor contributing to arrhythmogenesis [5].

Many other factors can contribute to the triggering of ventricular arrhythmias in this setting. Scorpion venom is a complex mixture of basic proteins, peptides, and other minor components such as salts, sugars, serotonin, histamine, and hyaluronidases. These proteins and peptides interact with voltage-gated sodium and potassium channels of excitable cell membranes [6]. Also, myocardial ischemia has been reported to occur during this type of envenomation [7]. Together, these two factors could slow down cardiac conduction contributing to a unidirectional block, which is a crucial element for the establishment of the reentrant circuit and is the mechanism of different types of arrhythmias including ventricular tachycardia.

Secondary disturbances such as hypokalemia and hyperglycemia may participate in this process. Probably, the massive catecholamine release is responsible for both events. In the first situation, sympathetic overstimulation causes ion influx into the cell (translocation) leading to hypokalemia and may contribute to the prolongation of the QTc interval. We usually do not replenish potassium because the electrolyte level normalizes spontaneously after cessation of the autonomic storm. In the second situation, catecholamines suppress insulin secretion, with consequent hyperglycemia and free fatty acid accumulation plus decreased glucose utilization. Murthy et al. [8] showed that insulin administration reverses the metabolic and electrocardiographic changes in acute myocarditis induced by Indian red scorpion venom in experimental dogs.

Izquierdo and Rodriguez [9] reported the case of a 12-year-old man who had a cardiac arrest due to pulseless ventricular tachycardia during envenoming by *Tityus pachyurus*. Bhadani et al. [10] reported the case of a 17-year-old man with cardiac arrest due to ventricular fibrillation after a red scorpion sting. There are approximately 1500 species of scorpions in the world; only 30 of them are dangerous and can cause severe envenomation [1]. Despite some slight differences, most dangerous scorpion stings cause similar clinical manifestations and theoretically all these species could induce severe ventricular arrhythmias during envenomation.

All of the above considerations support the idea that life-threatening arrhythmias such as ventricular tachycardia and ventricular fibrillation can occur during scorpion envenomation and could cause sudden death of the victims. Scorpion envenomation could be responsible for a percentage of cases of sudden death, mainly of children, in which cardiac structural disease or congenital channelopathies were not identified.

There are many other reports of cardiac complications occurring after scorpion stings in children, including acute pulmonary edema and cardiogenic shock [4, 11]. On the other hand, there are few reports of severe manifestations in adults.

The severity of envenomation seems to depend on body weight, with the smaller body volume of children possibly explaining the stronger action of scorpion venom in these patients [12]. Furthermore, other factors can contribute to important systemic manifestations, such as the amount of venom found in the scorpion, the type of scorpion, and the sensitivity of the victim. Because of these factors, severe cases also can occur among adult patients.

In our case, the animal responsible for this incident was not definitely identified. Lack of identification of the scorpion is the major limitation of this case report. However, many of the arguments presented below indicated that a scorpion was the most plausible animal responsible for the manifestations observed. Clinical and laboratory presentation, including important pain without inflammatory signals at the site of the sting, symptoms of profuse emesis and sweating, hyperglycemia associated with hypokalemia, hyperamylasemia, and the fact that the incident occurred in an area infested with scorpions and that the patient's clinical condition improved a lot after scorpion antivenom administration, further supports our conclusion. In our region, only a spider incident caused by *Phoneutria sp.* could have a similar presentation because the venom of this spider is closely similar to that of scorpions, although it rarely causes the severe manifestations reported here. Three dangerous scorpion species are found in our country: *Tityus serrulatus*, *Tityus bahiensis*, and *Tityus stigmurus*, although only this first specie is observed in our region [5].

Regarding treatment, administration of scorpion antivenom, which only neutralizes circulating toxin, is recommended in moderate and severe cases. Although its efficacy and benefit are controversial, this antivenom is more efficient when given early. In the present case we observed a general improvement of patient condition after administration [1, 6]. Our patient received the antivenom about two hours and thirty minutes after the incident, a period considered to represent a good time window for treatment.

Complete recovery of cardiac function was observed after one month in our case, but we do not know if after this event the patient will have a higher risk of cardiac complications such as development of heart failure or cardiac arrhythmias in the future.

## 4. Conclusion

Life-threatening arrhythmias such as sustained ventricular tachycardia can be observed during some types of scorpion envenomation. These severe arrhythmias could be responsible for some cases of sudden death of unclear etiology in regions where these incidents are frequent.

## Figures and Tables

**Table 1:** Laboratory tests on admission and 24 hours later.

Laboratory tests	Admission	24 hours	Normal range
Glycemia (mg/dL)	418	136	65–99
Potassium (mmol/L)	2.6	3.4	3.5–5.0
Ionized calcium (mmol/L)	1.09	1.11	1.12–1.32
Sodium (mmol/L)	135	131	135–145
Lactate (mmol/L)	9.1	2.6	0.5–2.0
Arterial pH	7.32	7.43	7.35–7.45
Base excess	−13.0	−0.9	−3.0 to +3.0
Bicarbonate (mEq/L)	12	22.8	25–35
PCO_2_ (mm Hg)	24	35	35–45
PO_2 _ (mm Hg)	77	112	75–100
PO_2_/FIO_2_	256	376	>300
Hemoglobin (g/dL)	15.6	13.2	12.5–15.5
White cell count (cell/mm^3^)	35.300	16.500	3.500–10.500
CK-MB (U/L)	33	46	<25
Amylase (U/L)	243	98	<125
Troponin I (mcg/L)	2.22	—	<0.01
